# Evolution Law of Concrete Interface Stress of Rigid-Frame Arch under Construction and Its Impact on Ultimate Load-Bearing Capacity

**DOI:** 10.3390/s23156868

**Published:** 2023-08-02

**Authors:** Yonghui Fan, Chao Luo, Yin Zhou, Ligui Yang, Xinglin Li, Jinlong Liao

**Affiliations:** State Key Laboratory of Mountain Bridge and Tunnel Engineering, Chongqing Jiaotong University, Chongqing 400074, China; fyh1995@mails.cqjtu.edu.cn (Y.F.); yin_zhou935215@163.com (Y.Z.); yangligui@cqjtu.edu.cn (L.Y.); 622220970001@mails.cqjtu.edu.cn (X.L.); jinlong_liao@mails.cqjtu.edu.cn (J.L.)

**Keywords:** fiber-optic strain sensors, rigid-frame arch bridge, stress evolution, interface between rings and intersegment, ultimate load-bearing capacity

## Abstract

To study the evolution of stress on the ring and segment interfaces during the construction process of the concrete encapsulation of the main arch ring in a rigid-frame arch bridge, alongside its impact on the ultimate load-bearing capacity of the main arch ring, a 1:10 scale model experiment was conducted by taking the 600 m Tian’e Longtan Bridge as the prototype. The key cross-section concrete strain data were collected during the entire construction process of the main arch ring via fiber-optic strain sensors, which were used to investigate the stress evolution at ring and segment interfaces. ANSYS APDL was employed to simulate the ultimate bearing capacity under various loading conditions of two different finite element models, which were, respectively, formed segmentally and by single pouring. The results revealed that (1) after the closure of the concrete encapsulation of the main arch ring, the concrete stress in the cross-section exhibits significant stress disparities. At the same cross-section, the level of the web concrete stress can reach 76% of the floor concrete stress, while the roof concrete stress level is less than 20% of the floor concrete stress. (2) At the junction of two adjacent work planes, there are considerable differences in the stress levels of the concrete on both sides. After the closure of the main arch ring, the intersegment stress ratios of the floor, web, and roof concrete are 60~70%, 40~60%, and 0~5%, respectively. (3) Loading conditions remarkably affected the ultimate bearing capacity of the main arch ring. Under mid-span loading and 1/4 span symmetrical loading conditions, compared to single-pour concrete encapsulation, the ultimate bearing capacity of the main arch ring with concrete encapsulated by segmented and ring-divided pouring decreased by 19.16% and 5.23%, respectively, compared to single-pour concrete encapsulation. This suggests that the non-uniformity of stress distribution in the concrete sheath can lead to reductions in the ultimate bearing capacity of the arch ring.

## 1. Introduction

The rigid-frame arch bridge is an advanced structural design featuring a strong steel truss as its backbone, whose concrete is poured into internal tubes and then covered with externally cast concrete in ring-segmented patterns [1]. Such a structure recently attracted increasing attention owing to its increased load-bearing capacity, attractive appearance, and lower long-term maintenance costs [2,3]. According to data collected, for rigid-frame arch bridges with a main span exceeding 300 m, casting the main arch ring’s external concrete is typically performed in relation to segmented rings, also known as the multi-working planes balanced casting method [4]. This construction method results in significant temporal disparities in structural loading between the floor concrete, the web concrete, and the roof concrete of the main arch ring’s external concrete, leading to substantial stress differences and compromising the load-bearing performance of the structure [5,6,7].

Comprehensive research on the stress evolution laws at the interfaces between the floor, web, and roof concrete of the main arch ring (hereafter referred to as inter-rings interface) and adjacent work plans interfaces (hereafter referred to as intersegments interface), as well as the influence of stress distribution on the ultimate load-bearing capacity, is scientifically and practically significant. It aids in clarifying the transmission mechanism of concrete at different ages and stress levels and optimizing the loading state of the main arch ring.

Currently, scholarly studies on the stress evolution during the construction process of external concrete encapsulation of the main arch ring in a rigid-frame arch bridge and its influence on the structural ultimate load-bearing capacity can be categorized into three types.

The first category focuses on the impact of the longitudinal segments’ quantity and segmentation location of the external concrete encapsulation on the structural stress [8,9]. These studies mainly use numerical simulation methods to compare the structure’s stress levels under different concrete encapsulation settings. For instance, Tong et al. [10] used the Nanpan River Bridge as an example and investigated the effect of the casting sequence of the longitudinal external concrete encapsulation on the arch ring structure’s instantaneous and permanent stresses based on numerical analysis. Similarly, Yang et al. [11] employed the Beipanjiang Bridge as a representative instance to conduct a comparative analysis of diverse exogenous concrete construction methodologies, distinctly from the vantage points of horizontal ring segmentation and longitudinal partitioning, whose study focused on the force-bearing state of the arch ribs during the execution of the construction process and the corresponding controlling determinants. Lin et al. [12] studied the process of pouring exogenous concrete for the rigid frame of the main arch ring using the six working plane method and the combination method of inclined stay cable plus multiple working planes. They analyzed the patterns of instantaneous stress and permanent stress changes when the two methods were applied during construction. Such research primarily focuses on summarizing patterns through simulation results under different construction plans without proposing measures to reduce the stress levels of the structures.

The second research category optimized the concrete encapsulation construction scheme based on controlling the stress and the line shape of the main arch ring [13,14,15]. For example, Au et al. [16] used the influence matrix method to adjust the cable force, controlling the stress during the construction process of the concrete encapsulation of the main arch ring within a reasonable range. Lin et al. [17] proposed a casting method for the main arch ring concrete, which sets four work plans symmetrically along the entire arch. This method adjusts the casting length and sequence of the concrete by fitting a continuous function on the ascending and descending sections of the control stress process line, thereby reducing the instantaneous stress of the rigid frame. Tong et al. [18] focused their study on a rigid-frame arch bridge with a main span of 600 m by comparing the stress and deformation results of the main arch ring obtained from Midas Civil computations, and they optimized the segmented ring-by-ring concrete encapsulation scheme for the main arch ring of this bridge. However, these studies optimize the structural stress from the perspective of algorithms and simulations without delving into the impact of stress reduction on the force-bearing performance of the structure.

The third category of research investigates the influence of various factors on the stress-bearing performance of the main arch ring from the perspective of limit-bearing capacity [19,20,21]. For example, Liu et al. [22] conducted a 1:16 scale experiment based on the Washiwo Bridge with a span of 95 m, studying the failure modes of catenary arches under the loading conditions of the arch crown and 1/4 span. Wang et al. [23] proposed a double-layer corrugated steel–concrete composite arch structure and studied its bearing capacity through a single-point loading experiment at the mid-span. Yang et al. [24] studied the change in the structure’s limit load-bearing capacity after the arch bridge’s reinforcement with ultra-high-performance concrete (UHPC). Hu et al. [25] studied the impact of the initial defects, such as the main arch ring’s fabrication and construction errors, on the structure’s ultimate load-bearing capacity. This type of study was mainly concentrated on the influence of factors such as arch axis line shape, structural form, materials, and initial defects on the bearing performance of the structure, with less research focusing on the stress state after construction completion.

In summary, the majority of studies focusing on the stress evolution of the main arch ring tend to spotlight the summary of patterns. Although some scholars have proposed stress optimization methods, most of them rely heavily on numerical simulations and accomplish their research through trial-and-error selection, lacking experimental support from measured data. Furthermore, the research regarding the impact of the stress state of the main arch ring after construction completion on bearing performance remains a gap in the literature.

Focusing on the dual issues of the lack of experimental data support for the stress evolution rules during the construction process of the main arch ring and the absence of studies on the impact of structural stress states on the ultimate load-bearing capacity, a 1:10 scale model experiment of the main arch ring was conducted based on the world’s largest rigid-frame arch bridge—the Tian’e Longtan Bridge—with a main span of 600 m. This 1:10 scale model has surpassed similar scale tests conducted on bridges worldwide previously [26,27]. ANSYS APDL was utilized to establish a finite element model of the bridge, and the model was validated by comparing the measured stress of the concrete at the key cross-sections and the simulation results. Ultimately, by comparing the ultimate load-bearing capacity using different construction methods as well as under different loading conditions, the influence of the structural stress state on the ultimate load-bearing capacity after the completion of the main arch ring construction is examined.

## 2. Engineering Example

**Figure 1:**
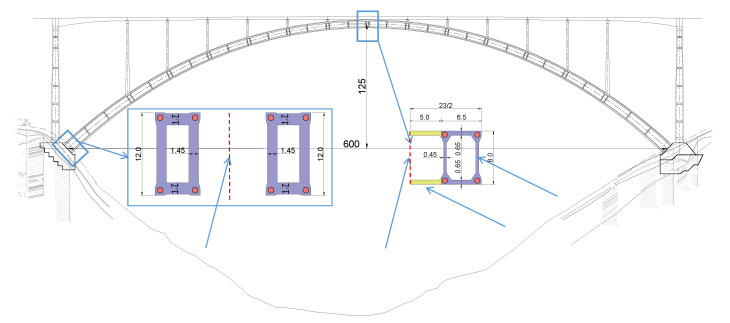
Elevation view of a concrete-filled steel tube stiff skeleton arch bridge (unit: m).

**Figure 2:**
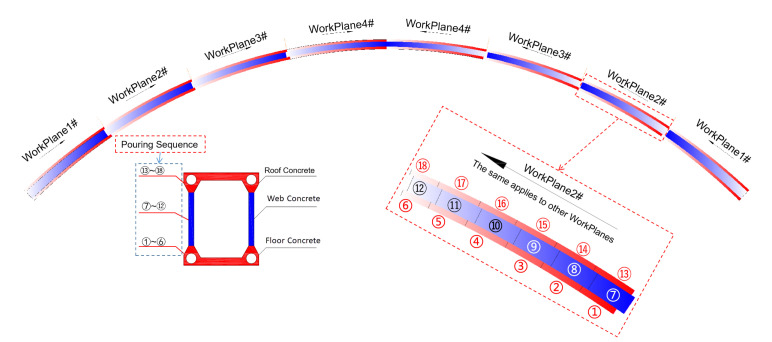
Schematic of segmental and ring-based concrete encapsulation on the primary arch ring.

The Tian’e Longtan Bridge, with a main span of 600 m and a height of 125 m (height-to-span ratio = 1/4.8), is located within Guangxi Province, China. The main arch is composed of three parts: a rigid steel skeleton, concrete-filled steel tube, and the external concrete forming a box-shaped cross-section; the size information of the external concrete at different sections is shown in Figure 1 [28]. The rigid steel skeleton adopts a Q420 material and is designed as a steel truss arch structure. During construction, it is installed using the cable-stayed pulling and hanging method. The concrete-filled steel tube is made of C80 concrete, and the construction process involves pumping through a lift-up pouring method. The external concrete uses C60 concrete and is constructed using the multi-working planes balanced casting method, as shown in Figure 2, the red part represents the floor and roof concrete, and the blue part represents the web concrete, the colours from deep to light and numbers both represent the sequence of concrete pouring. In the transverse direction, the external concrete is divided into three rings: the floor, web, and roof concrete. In the longitudinal direction, each concrete ring is further divided into eight working planes, and each working plane is poured multiple times to achieve a balanced pouring.

## 3. Model Test Methodology

It should be pointed out that the load effect will vary with the change in scale, and a reasonable scale can improve the accuracy of inferring the original bridge state from the model test results. In addition, a smaller scale can lead to a smaller structure size, increasing the difficulty of welding the steel skeleton and the risk of pipe blockage during the pouring process of concrete inside the pipe. Additionally, thinner concrete will reduce the thickness of the protective layer of the steel bar. On the contrary, a larger scale will increase the demand for experimental space in the experiment. Considering multiple factors such as the load effect, availability of materials, the feasibility of steel structure fabrication, experimental space, and structural safety during construction, a scale of 1:10 was chosen for this experiment.

In accordance with the principle of stress equivalence [29,30], a main arch ring model experiment with a main span of 60 m was conducted. In order to minimize the influence of scale effects [31], strict scaling ratios were used in the geometric design, including overall dimensions such as span, the height of mid-span position, and linear forms. In terms of materials, a substantial number of concrete mix ratio experiments were carried out to ensure that the concrete of the model arch and the original bridge were essentially identical in parameters such as material strength and fluidity [32].

**Figure 3:**
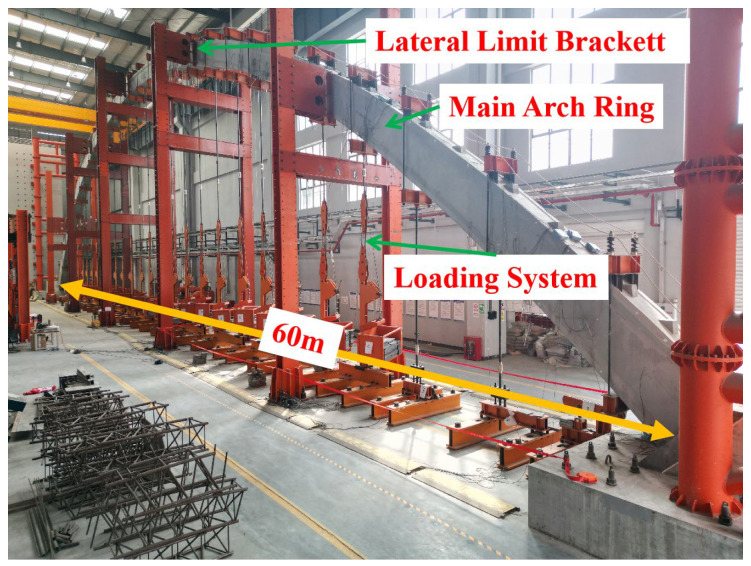
Picture of the model test.

Notably, due to spatial constraints in the laboratory, the model experiment focused on a single arch ring. This focus made the issue of lateral stability of the main arch ring more prominent. To enhance the safety of the structure during construction, a limiting bracket along the longitudinal direction of the bridge every 10 m was arranged, as shown in Figure 3.

To ensure stress equivalence between the model and the original bridge, the construction process required nine times the self-weight of the completed structure to be applied as a permanent load at the corresponding load points. The model arch is set with a loading point every 2 m along the longitudinal direction of the bridge, and a total of 29 loading points are set for the entire bridge. To enhance the local stability near the loading points, transverse partitions with a thickness of 50 mm are also arranged every 2 m along the length of the bridge [28]. During the casting process of the external concrete, the construction method involved using the permanent load balance weight before casting the concrete.

**Figure 4:**
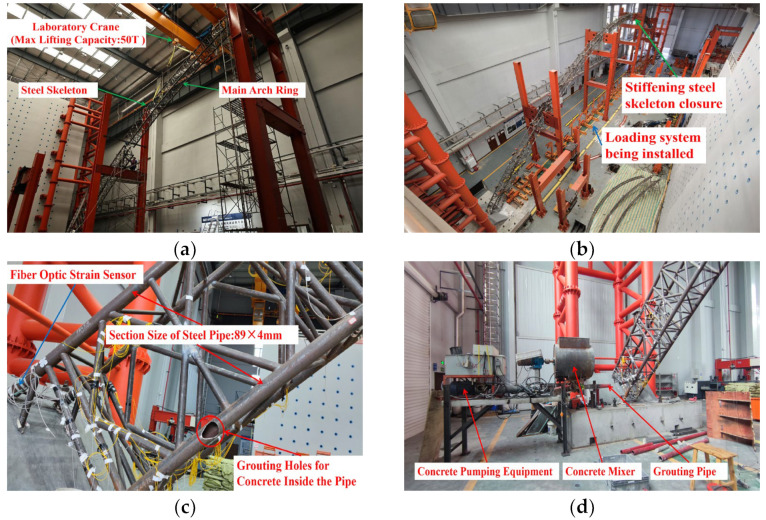

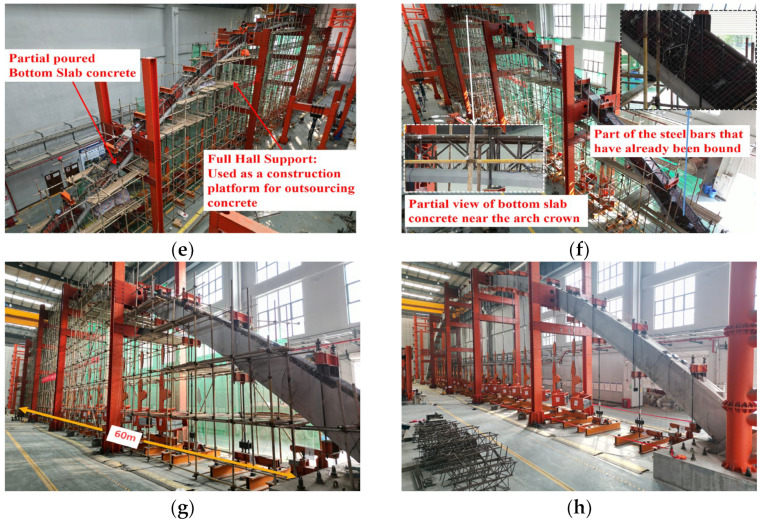
Display diagrams of the vital construction stages of the main arch ring of the model bridge. (**a**) Install the rigid steel skeleton using the large section lifting method; (**b**) closure of rigid steel skeleton (construction stage 1#); (**c**) concrete inlet in the pipe; (**d**) concrete grouting equipment inside the pipe (construction stage 2#); (**e**) layout of full framing; (**f**) closure of the floor concrete (construction stage 16#); (**g**) closure of the web concrete (construction stage 28#); (**h**) closure of the roof concrete (construction stage 40#).

Considering the primary arch ring segmentation, the external concrete was cast in 18 stages. Considering the erection of the rigid steel skeleton, the pouring of inner tube concrete, and the application of the permanent load balance weight, the primary arch ring was divided into 40 construction phases, as illustrated in Table 1. Figure 4 shows pictures of some key construction nodes of the primary arch ring of the model bridge.

## 4. Strain Testing Scheme for Externally Wrapped Concrete

The strain testing of externally wrapped concrete includes inter-ring strain tests at the 17 evenly distributed cross-sectional locations and strain tests near the section locations of the working plane of the concrete.

### 4.1. Concrete Inter-Rings Interface Strain Testing Scheme

**Figure 5:**
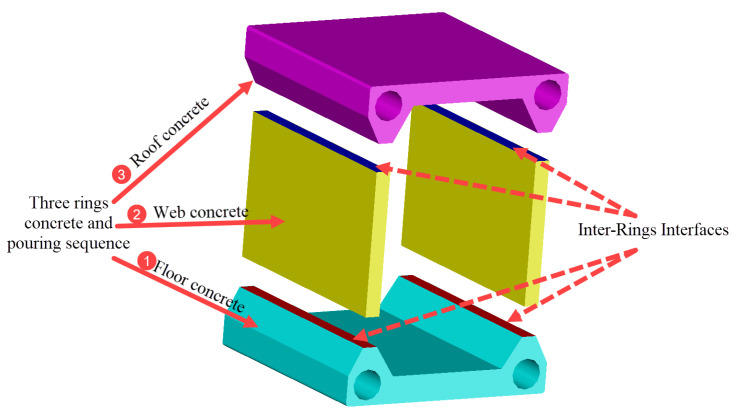
Schematic diagram of the force transfer surface and pouring sequence for the concrete rings wrapped around the main arch ring.

**Figure 6:**
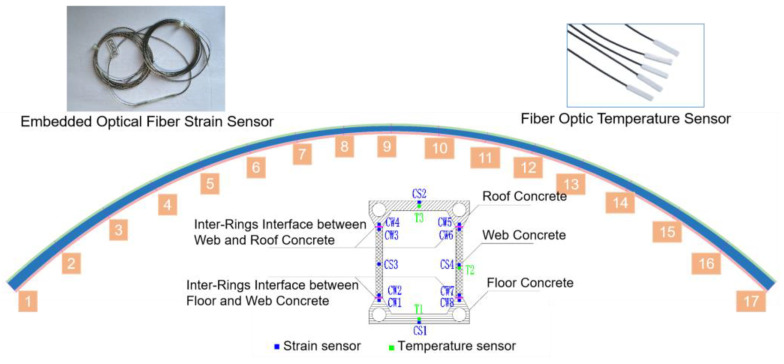
Diagram of the stress testing scheme for the externally wrapped concrete ring.

The inter-rings interface refers primarily to the junction of the floor, web, and roof concrete, forming the three rings of the concrete structure, as depicted in Figure 5. Due to the varying sequence of pouring these three rings of concrete, their participation in the overall structural load-bearing varies, resulting in an uneven distribution of stress levels in these three rings of concrete after the primary arch ring is completed. This heterogeneity differs significantly from the stress distribution of arch rings formed through a single pouring operation and directly impacts the load transmission within the arch concrete, potentially reducing the limit load-bearing capacity of the arch rings below the original design value. Consequently, it is necessary to monitor the strain of the abovementioned panels throughout the construction process to capture the differences in interface stress [33,34]. Based on the model experiment, longitudinal strain sensors are evenly placed at 17 points along the span of the main arch ring specimen, as shown in Figure 6.

In Figure 6, 17 sections for strain testing were evenly distributed along the longitudinal direction of the main arch ring. Each section was equipped with 15 fiber-optic strain sensors [35] located at varying heights on the 17 sections; the relevant parameters of the sensor are shown in Table 2. Sensors CS1–CS4 are embedded and used to collect strain data of the floor, web, and roof concrete, while CW1–CW8 were used for testing strain data near the inter-rings interface between the floor and web concrete, and the inter-rings interface between the web and roof concrete.

The structural strain can be calculated using Equation (1) and based on sensor wavelength data:

(1) $$\varepsilon =\left(l_{c}-l_{0}\right)c_{w}\times 10^{3}$$

In the equation, $l_{0}$ represents the initial wavelength at the time of sensor installation, $l_{c}$ represents the current wavelength of the fiber-optic sensor, and $c_{w}$ is the wavelength conversion coefficient, which is taken according to Table 2. ε represents the total strain that occurs at the current moment of the structure relative to the time when the sensor is installed.

To eliminate the influence of environmental temperature during the construction process on strain data, temperature sensors T1–T3 are embedded within the externally wrapped concrete, evenly distributed at three section heights, and the corresponding calculation method is shown in Equation (2):

(2) $$\varepsilon_{f}=\varepsilon -\left(l_{c}-l_{0}\right)c_{w}\alpha_{t}\times 10^{3}$$

In the equation, for this experiment, $\varepsilon_{f}$ represents the strain of the structure at the measurement point position at the current moment relative to the sensor installation time under self-weight and counterweight load, and $\alpha_{t}$ represents the linear expansion coefficient of the structure.

### 4.2. Intersegments Interface Strain Testing Scheme

**Figure 7:**
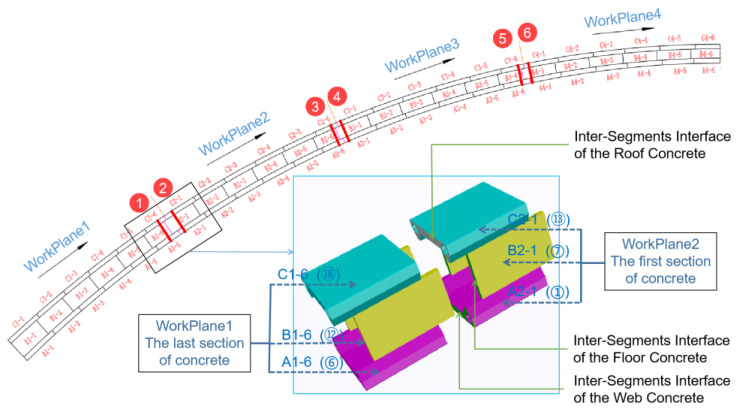
Distribution diagram of sectional interfaces in externally wrapped concrete.

The intersegments interface primarily refers to the interface between two adjacent concrete sections within the same ring. When constructing externally wrapped concrete, a multi-plane balanced pouring method was used, and there is a particular age difference between the concrete on both sides of the intersegments interface. This age difference reaches its maximum when the interface is located between two adjacent working planes. To some extent, this difference reflects the timing of the concrete’s participation in structural loading and the duration of stress accumulation; hence, the stress difference between two sections of concrete often correlates positively with the age difference. Based on these considerations, three intersegment interfaces were selected along the main arch ring in the longitudinal direction. Two test sections were set up on either side of each interface, as shown in Figure 7.

**Figure 8:**
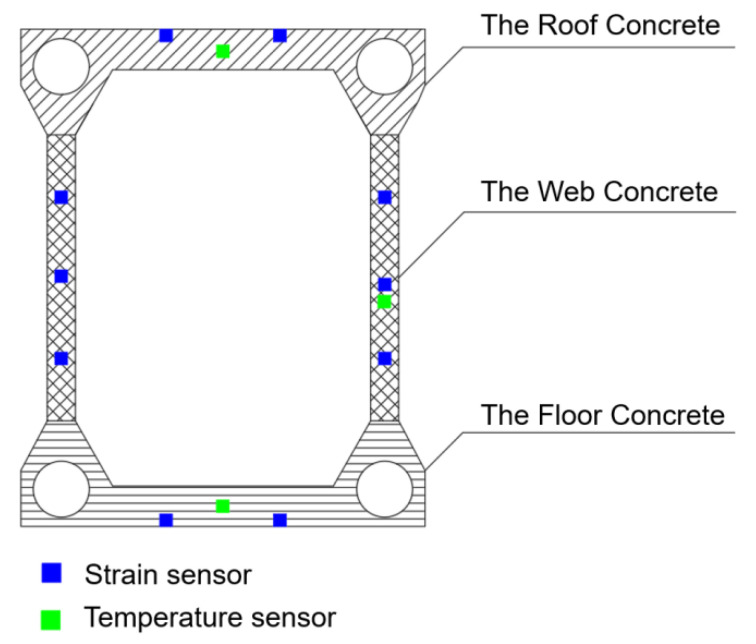
Schematic diagram of sensor layout at sectional interfaces.

In Figure 7, the numbers encircled in brackets (➀, ➅, ➆, ⑫, ⑬, and ⑱) represents the concrete pouring sequence, and the A*i*-*j*, B*i*-*j*, and C*i*-*j* (*i* =1,2,3,4; *j* =1,2, …,6) respectively represent the concrete in the *j*th part of the *i*th workplane of the floor, web, and roof concrete. The three intersegments interfaces were located, respectively, at the junctions between work planes 1 and 2, 2 and 3, and 3 and 4. A test section, numbered from 1 to 6, was set up for each interface, with the sensor layouts for each section shown in Figure 8.

In Figure 8, each cross-section is equipped with 15 fiber-optic sensors. Among these, sensors CS1–CS2, CS3–CS4, and CS5–CS10 were strain gauges used to gather stress data from the concrete structure’s roof, floor, and web. To eliminate the potential influence of environmental temperature and exothermic hydration reactions within the concrete on the strain data, temperature sensors T1–T3 were strategically positioned at three different heights along the intersegment interfaces, following the same arrangement as in the case of the annular space. The configuration allowed for accurate thermal correction and ensured the strain measurements’ integrity.

## 5. Interfacial Stress Evolution in the Encapsulated Concrete Annular Space

### 5.1. Intersegments Interface Stress Evolution

**Figure 9:**
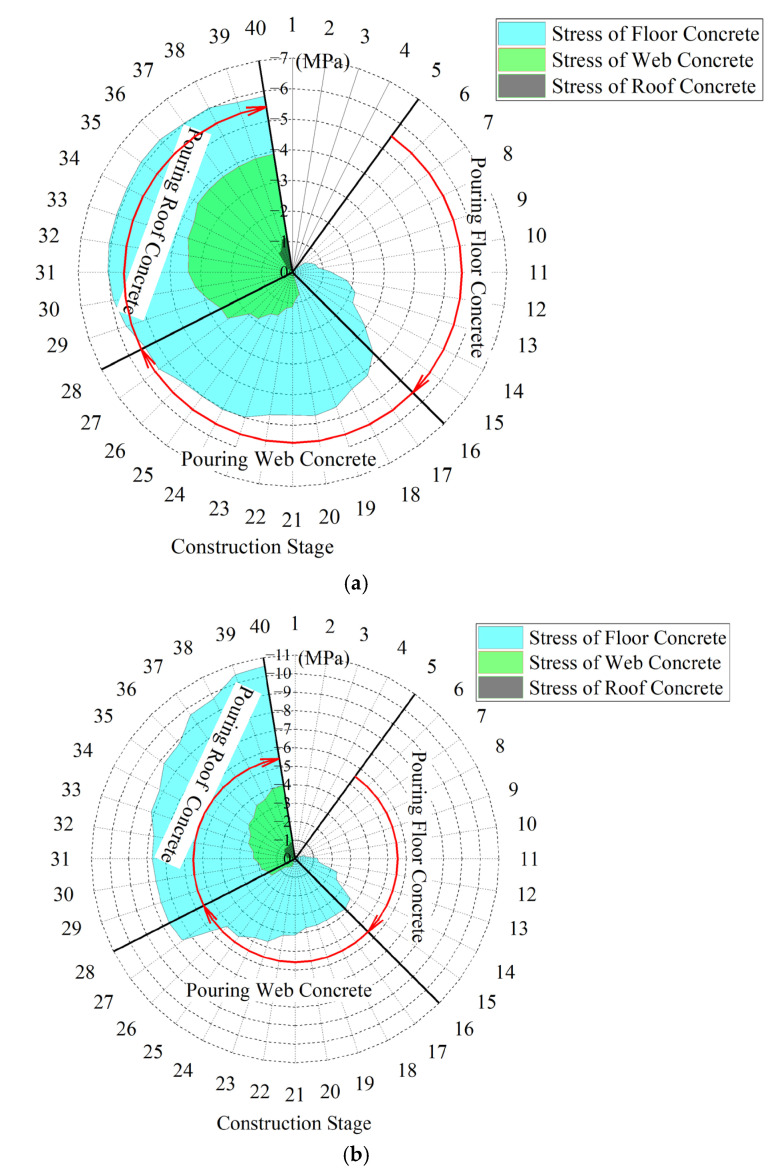

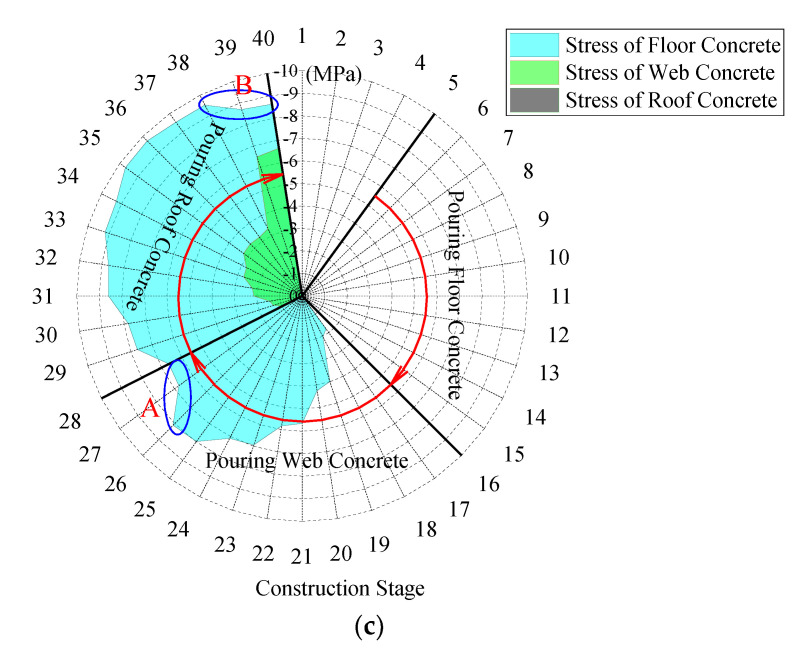
The stress curve of each main section in the construction stage of the main arch ring. (**a**) Section of arch foot; (**b**) 1/4 span section; (**c**) mid-span section.

To quantitatively analyze the evolution of interfacial stress in the main arch ring during the construction process of the encapsulated concrete, Hooke’s Law was used to calculate the stress in the arch foot section, ¼ span section, and mid-span section. The stresses in the floor, web, and roof concrete at various construction stages were extracted from these sections. The derived stress profiles are illustrated in Figure 9.

Figure 9 shows that the stress level in the tri-ring concrete generally presented a pattern where the floor concrete bore the highest stress, followed by the web concrete, and the roof concrete had the least stress. After the encapsulated concrete was poured, the stress ratios of the tri-ring concrete in the arch foot section, 1/4-span section, and mid-span section were 1:0.67:0.20, 1:0.38:0.08, and 1:0.76:0.02, respectively. In Figure 9a,b, as the construction stage advances, the compression stress level of the tri-ring concrete exhibited an overall upward trend. Each ring’s compression stress developed rapidly before the current ring closure. After the closure, it formed a unified structure with the rigid steel frame and participated in the overall structural load, which subsequently slowed the stress development. In Figure 9c, the compressive stress of the floor concrete at the mid-span position decreases from 8.10 MPa to 6.64 MPa (Area A in Figure 9c) during the web concrete closure stage and 9.51 MPa to 8.62 MPa (Area B) during the roof concrete closure stage, decreasing by 18.02% and 9.36%, respectively. This reduction was due to the increased negative moment at this location caused by the poured concrete in the web and roof concrete near the arch crown, thereby reducing the compressive stress in the floor concrete. Furthermore, as the moment of inertia at the arch crown section gradually increased with the closure of each ring of concrete, the negative moment’s effect in reducing the compressive stress in the floor concrete was more pronounced in the early stages of construction.

**Figure 10:**
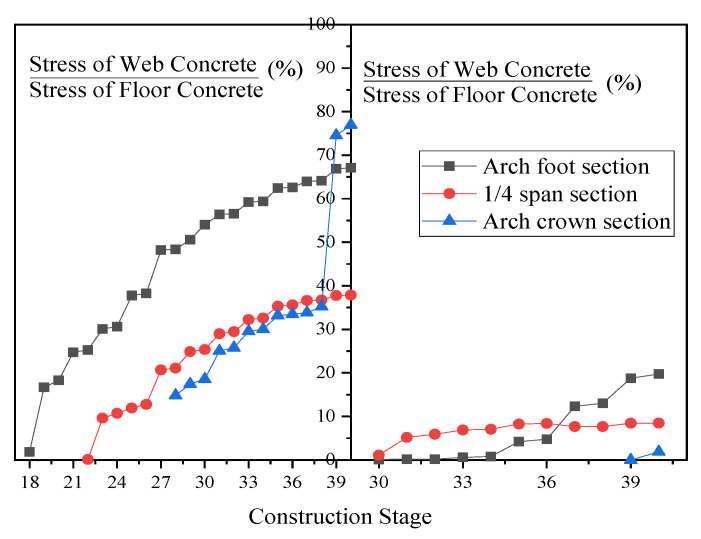
Three-ring stress ratio diagram of concrete encased in the main arch ring.

To understand the changes in the relative stress state of the tri-ring concrete during the construction process of the main arch ring, we have calculated the stress ratios of the tri-ring concrete in the arch foot section, ¼ span section, and arch crown section during the construction process, as shown in Figure 10.

In Figure 10, all cross-sections present a characteristic where the stress level of the three rings’ concrete gradually converged as the construction stage progressed. This was mainly manifested as an overall increasing trend in the ratio of the web concrete stress to the floor concrete stress and the roof concrete stress to the floor concrete stress at the same cross-section. After the closure of the main arch ring, the maximum web concrete stress at the same cross-section could reach 76.94% of the floor concrete, while the roof concrete accounted for only 19.73%. It should be pointed out that for the arch foot cross-section, the ratio of web concrete stress to floor concrete stress increased rapidly at the 19# construction stage. This was due to the large inclination angle of the arch axis and the horizontal plane at this location, resulting in a larger axial component of the self-weight load of the encapsulated concrete along the main arch ring.

However, when pouring the roof concrete, there was no similar phenomenon as in the web concrete. There were two main reasons for this. Firstly, the roof concrete had a smaller volume than the web concrete, which resulted in a smaller load component along the arch axis due to the self-weight of the former. Secondly, after the roof concrete of the arch foot cross-section hardened, it participated together with the main steel tube and the concrete within the tube in the local load-bearing of the structure. They jointly bore the axial component of the self-weight load from the newly poured roof concrete.

### 5.2. Analysis of Intersegments Interface Stress Evolution

**Figure 11:**
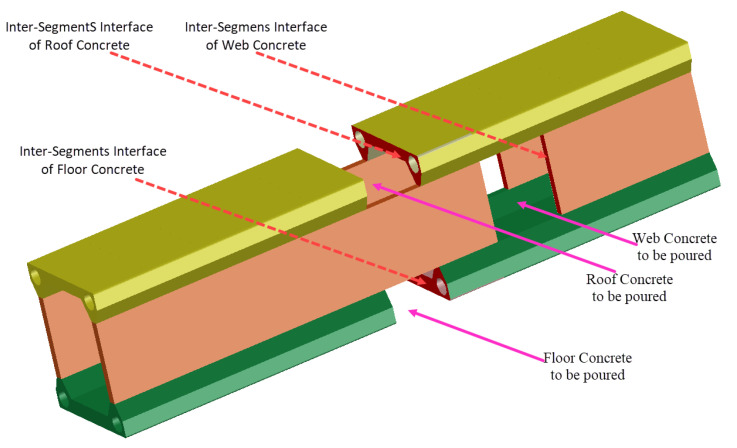
Schematic diagram of force transmission position between concrete sections wrapped around the main arch ring.

During the pouring of the encapsulated concrete in the main arch ring, each ring was divided into multiple work surfaces in the longitudinal direction, and each work surface was completed in multiple stages of pouring. Figure 11 is a schematic diagram showing the location of the intersegment force transfer surfaces in the encapsulated concrete of the main arch ring. Before the closure of each ring of concrete, the axial forces between the work surfaces cannot be directly transmitted. Once a ring of concrete achieves closure and forms strength, the load-bearing capacity and stability of the main arch ring are enhanced. The axial forces generated in subsequent construction stages can be transmitted across work surfaces through the intersegment force transfer surfaces.

**Figure 12:**
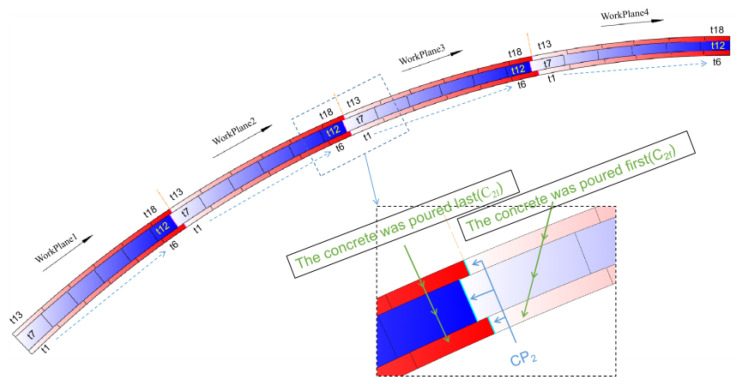
Construction sequence diagram of main arch ring wrapped with concrete.

The construction sequence of the encapsulated concrete is shown in Figure 12, the dashed arrow in the figure indicates the direction of concrete pouring. The pouring sequence of each segment of concrete is denoted as $t_{i}\left(i=1,2,3,\cdot \cdot \cdot ,18\right)$, and the interface between the *N*th and the (*N* + 1)th work planes of each ring is represented as $CP_{N}$ (N = 1, 2, 3). The concrete on either side of the interface is denoted as $C_{Nf}$ (concrete of the earlier poured segment) and $C_{Nl}$ (concrete of the later poured segment), according to the pouring sequence, and their respective stresses are denoted as $\sigma_{Nf}$ and $\sigma_{Nl}$. $P_{FN}$, $P_{WN}$, and $P_{RN}$ are used to represent the stress ratios of the concrete on either side of the intersegment interface in the floor, web, and roof concrete, respectively. Thus, we have:

(3) $$P_{FN},P_{WN},P_{RN}=\frac{\sigma_{Nf}}{\sigma_{Nl}},\left(N=1,2,3\right)$$

**Figure 13:**
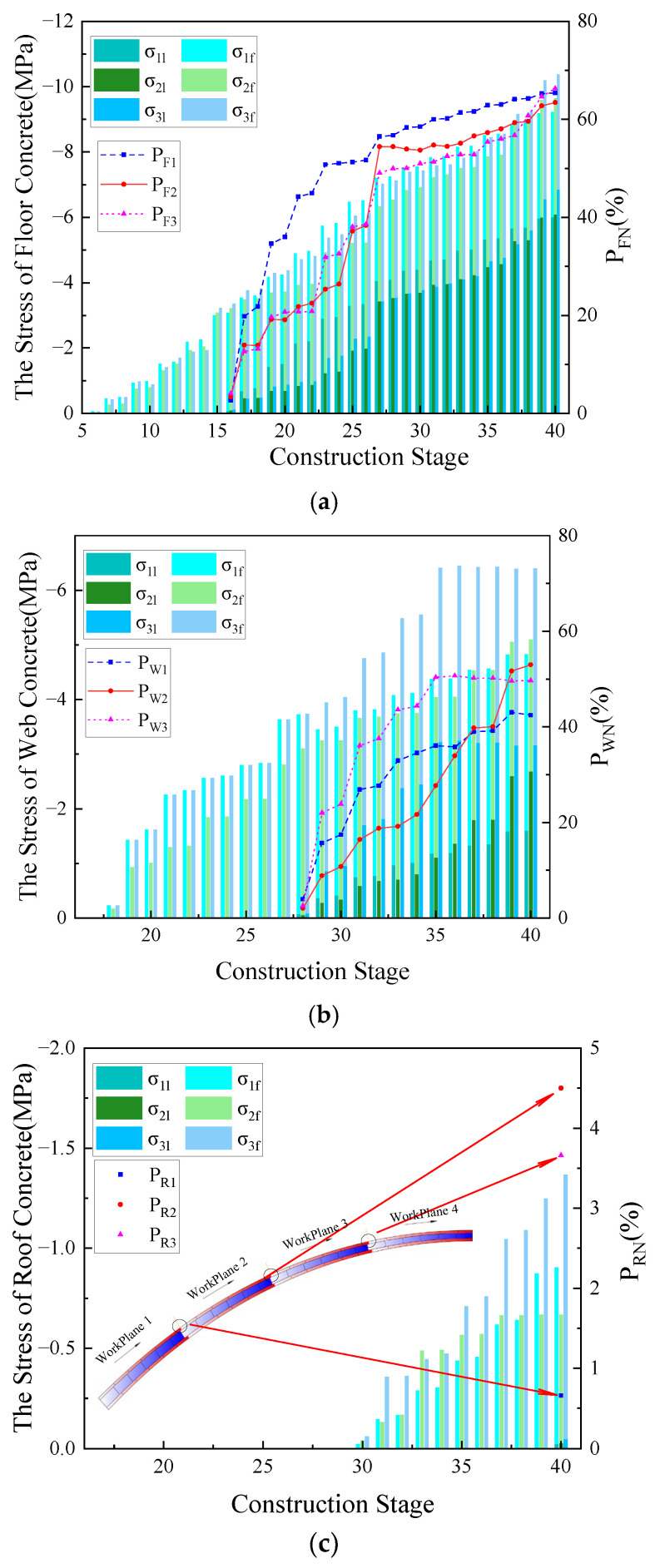
Concrete stress results of main arch ring wrapped with concrete in longitudinal section. (**a**) Stress results between concrete segments of floor concrete; (**b**) stress results between concrete segments of web concrete; (**c**) stress results between concrete segments of roof concrete.

The stress results for the intersegments interfaces during the construction process of the encapsulated concrete in the main arch ring have been collated, as illustrated in Figure 13.

As seen from Figure 13a,b, with the increase in the construction stage of the encapsulated concrete in the main arch ring, the intersegment stress ratio of the floor and web concrete gradually increases. After the construction of the encapsulated concrete was completed, the intersegment stress ratio $P_{FN}$ of the floor concrete is within the range of 60~70%, and $P_{F1}$, $P_{F2}$, and $P_{F3}$ are essentially equal; the intersegment stress ratio $P_{WN}$ of the web concrete is within the scope of 40~60%. Figure 13c circles the longitudinal segment positions during the pouring of the roof concrete. The intersegment stress ratios $P_{WN}$ for each segment are all less than 5%, which is significantly lower than the intersegment stress ratios of the floor and web concrete at this construction stage. This is because the pouring time of the roof concrete is later than that of the floor and web concrete, and the time it participates in structural load-bearing is also later. Compared with the floor and web concrete, the roof concrete lacked a process where the stress ratio increased with the construction stage. A large part of its stress was caused by its own gravitational load, and the mass of the roof concrete was smaller than that of the floor and web concrete, resulting in a smaller gravitational load. This eventually leads to the phenomenon where the intersegment stress ratio of the roof concrete is less than that of the first two rings of concrete.

## 6. Finite Element Model

### 6.1. Model Introduction

**Figure 14:**
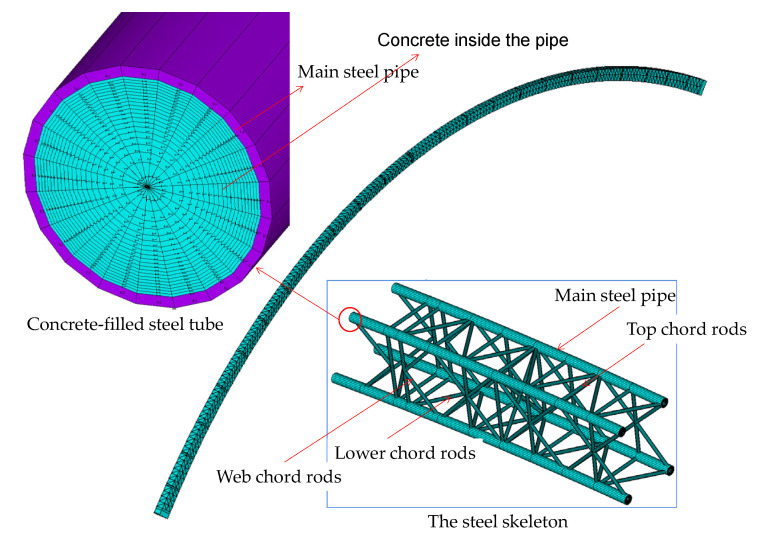
The finite model of main arch ring.

In the pursuit of a comprehensive study on the influence of uneven stress distribution in the external concrete ring and segmental joints, following the closure of the main arch of a rigid-frame arch bridge, a finite element model of the main arch ring has been constructed. This model utilizes the robust capabilities of the ANSYS APDL 18.0 [36], offering an extensive analysis of the structural behavior under different stress conditions. Figure 14 clearly represents the finite element model of the main arch ring.

Our approach, encompassing numerical simulation and advanced computational mechanics, aims to reveal potential vulnerabilities within the structural integrity of arch bridges. By focusing on the uneven distribution of stress after the arch ring closure, we can better comprehend its effects on the overall load-bearing performance of these structures. In turn, the acquired insights can significantly contribute to improving bridge design and safety strategies and provide a robust foundation for further academic studies in this field.

In Figure 14, the global coordinate system’s X, Y, and Z directions correspond to the main arch ring’s longitudinal, vertical, and lateral directions, respectively. The primary steel tubes, upper chord rods, lower chord rods, web rods of the rigid steel skeleton, and concrete-filled tubes are simulated using Beam188 elements. In contrast, the externally wrapped concrete is modeled using Shell181 elements. The complete bridge model comprises 6660 elements in total, including 3888 Beam188 elements and 2772 Shell181 elements. 

During calculations, displacement and rotation in all directions are restrained at every node on the surfaces where the arch feet are erected. Load conditions consist of the self-weight of the structure and the actual counterweights at the model bridge’s loading points. The construction process of the main arch ring is simulated using birth-and-death elements.

### 6.2. Finite Element Model Validation

**Figure 15:**
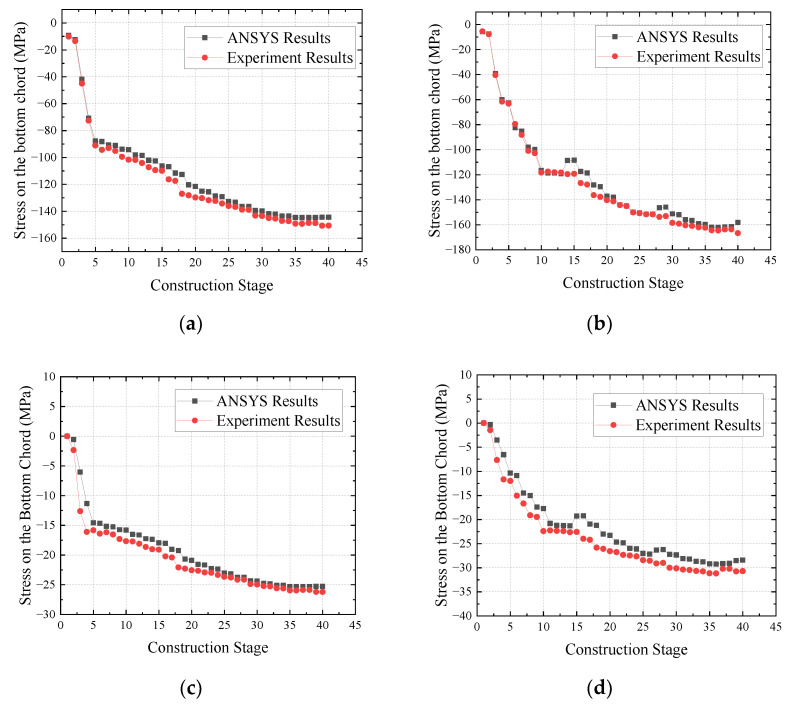

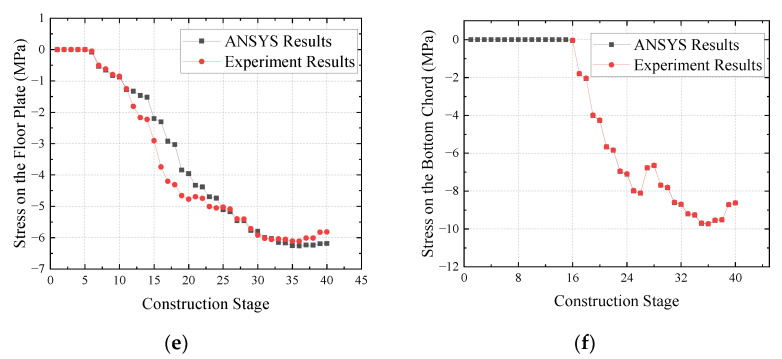
Comparison of simulated and measured results of each component throughout the construction process. (**a**) Stress results of the main steel pipe at the arch foot section; (**b**) stress results of the main steel pipe in the arch crown section; (**c**) stress results of concrete inside the arch foot section pipe; (**d**) stress results of concrete inside the arch crown section pipe; (**e**) stress results of floor concrete at arch foot section; (**f**) stress results of floor concrete at arch crown section.

To validate the correctness of the finite element model, we have compared the stress results measured and simulated in the rigid steel skeleton, the concrete inside the tubes, and the externally wrapped concrete at some key cross-sections during various construction stages. These comparisons are graphically presented in Figure 15.

Our comparison across multiple stages ensures a rigorous validation of our finite element model, thus enhancing its credibility and potential application for future research. This method facilitates a better understanding of the stress distribution and performance of different bridge elements, making a significant contribution to the overall field of structural engineering.

As illustrated in Figure 15, each component’s measured stress and simulation results during the construction process generally align well, exhibiting a consistent overall trend. Among them, the maximum relative error between the simulation results and experimental results for the main steel tube of the rigid steel skeleton throughout the construction process is 14.42 MPa, with the maximum relative error being 11.35%. These occur during the casting of the first segment of the web concrete stage, located at the top-chord main steel tube of the arch cross-section.

During the casting of externally wrapped concrete, the maximum relative error between the simulated and experimental results of the concrete inside the tubes is 4.09 MPa, which occurs during the casting of the third segment of the floor concrete stage. The maximum relative error between the simulated and experimental results of the floor concrete throughout the construction process is 1.44 MPa.

Throughout the construction process of the main arch ring, the stress results of the concrete inside the tubes and the externally wrapped concrete are significantly smaller compared to the main steel tube. Hence, the absolute error better represents the consistency between the ANSYS results and the experimental outcomes.

Overall, the error between the simulated and experimental results falls within an acceptable range. These comparative results substantiate the finite element model’s accuracy, thereby supporting the rationality of employing this finite element model for subsequent calculations.

## 7. Ultimate Load-Bearing Capacity Analysis

### 7.1. Introduction to the Computational Model

Experimental results demonstrate that when using a segmented ring-based construction method for externally concreted rigid-frame arch bridges, significant stress differentials exist between the tri-ring concrete segments and longitudinally adjacent concrete sections. In order to investigate the impact of this phenomenon on the structural performance of the main arch ring after its construction, we apply the ‘birth-and-death’ element method based on the validated finite element model in Section 6. This is achieved by adjusting the groups of activated elements under different construction stages, enabling a single pour for all the external concrete. This model, hereafter referred to as Model 2, demonstrates the construction sequence of the main arch ring, as shown in Table 3. The finite element model established following the conventional construction sequence will be designated as Model 1 for comparative purposes.

### 7.2. Ultimate Load-Bearing Capacity: Loading Scenarios and Results

**Figure 16:**
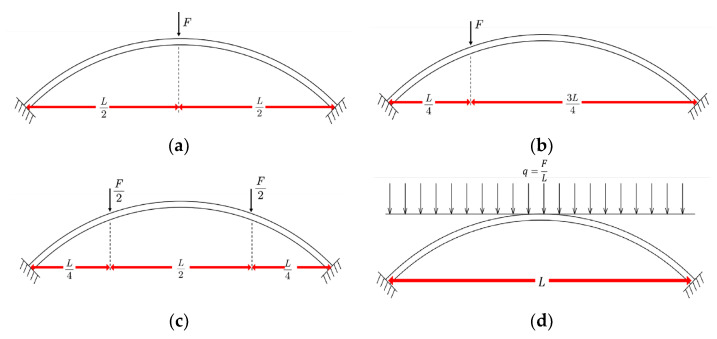
Schematic diagram of the loading conditions for the ultimate bearing capacity of the finite element model of the model bridge. (**a**) Single-point loading at the mid-span position; (**b**) single-point loading at 1/4 span position; (**c**) symmetrically loaded at 1/4 span position; (**d**) apply uniform load throughout the entire span.

In Model 2, the external concrete is activated simultaneously, meaning that all ringed and segmented concrete sections engage in structural stress simultaneously. After the completion of the sixth construction phase, the load-bearing distribution between and within the external concrete rings becomes more uniform, avoiding noticeable stress discontinuities. To quantitatively analyze the effect of uneven stress distribution between and within the rings of external concrete on the structure’s load-bearing performance, we referenced other scholars’ research methods in the arch bridge’s ultimate load-bearing capacity [37,38,39,40,41,42]. We separately calculated the ultimate load-bearing capacity of Models 1 and 2 under four loading scenarios: mid-span loading, quarter-span single-point loading, symmetric loading at quarter-span, and full-span uniformly distributed load (as shown in Figure 16). The calculations consider the impact of geometric non-linearity and material non-linearity. Gradual loading during calculations was implemented through time step increments, with each adjacent time step having a load increment of 2000 N. The calculation results are presented in Table 4.

**Figure 17:**
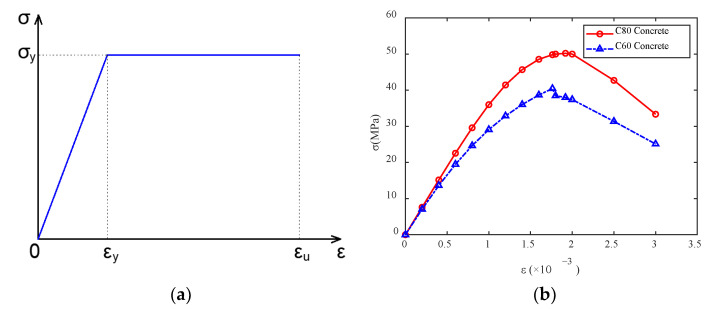
Stress–strain curve for main arch ring material. (**a**) Stress–strain curve for Q420 steel material; (**b**) stress–strain curve for concrete material.

The rigid steel frame of the main arch ring is made of Q420 steel. An ideal elastic–plastic model is used and simulated using the bilinear kinematic hardening model (BKIN) in the finite element model. As shown in Figure 17a, the relationship between δ and ε can be expressed using Equation (4):

(4) $$\sigma =\left\{\begin{array}{l} E\varepsilon ,\varepsilon \leq \sigma_{y}\\ \sigma_{y},\sigma_{y}<\varepsilon \leq \varepsilon_{u} \end{array}\right.$$

In the equation, *E* represents the elastic modulus of Q420 steel, with *E* = 2.06 × 10^5^ MPa; $\sigma_{y}$ represents the yield strength of the material, with $\sigma_{y}$ = 420 MPa; $\varepsilon_{y}$ represents the strain of the material when it reaches yield strength.

For the concrete material, the reference is “Code for Design of Concrete Structures” (GB50010-2010), [43]. It is assumed that both C80 and C60 concrete meet the following conditions:

(5) $$\sigma =\left(1-d_{c}\right)E_{c}\varepsilon$$

where $d_{c}$ is the parameter for uniaxial compressive damage evolution of concrete, which can be determined using Equation (6):

(6) $$d_{c}=\left\{\begin{array}{l} 1-\frac{\rho_{c}n}{n-1+x^{n}},x\leq 1\\ 1-\frac{\rho_{c}}{\alpha_{c}{\left(x-1\right)}^{2}+x},x>1 \end{array}\right.$$

In the equation, $\alpha_{c}$ is the parameter value of the descending segment of the uniaxial compressive stress–strain curve of concrete, while $\rho_{c}$, n, and x can be determined using Equations (7) to (9):

(7) $$\rho_{c}=\frac{f_{c,r}}{E_{c}\varepsilon_{c,r}}$$

(8) $$n=\frac{E_{c}\varepsilon_{c,r}}{E_{c}\varepsilon_{c,r}-f_{c,r}}$$

(9) $$x=\frac{\varepsilon }{\varepsilon_{c,r}}$$

In these Equations, $f_{c,r}$ represents the representative value of the uniaxial compressive strength of the concrete; $\varepsilon_{c,r}$ indicates the peak compressive strain of the concrete corresponding to $f_{c,r}$. Ultimately, the stress–strain curves for the C80 concrete inside the main arch ring and the C60 concrete encasing are obtained, as shown in Figure 17b.

Table 4 shows a significant difference in the ultimate load-bearing capacity of the main arch ring under different loading conditions. The ultimate load-bearing capacities of both Model 1 and Model 2 do not exceed 100 tons when loaded at the mid-span. However, under uniform loading conditions, the ultimate load-bearing capacities of both models exceed 1000t, indicating a significant influence of loading conditions on the ultimate load-bearing capacity post-arch ring closure. In addition, with the exception of the 1/4 span single-point loading condition, the ultimate load-bearing capacity of Model 1 under all other loading conditions is less than Model 2. Compared to Model 2, the ultimate load-bearing capacities of Model 1 under mid-span and ¼ span symmetric loading conditions decreased by 19.16% and 5.23%, respectively. This suggests that an uneven stress distribution in the encased concrete can reduce the arch ring’s ultimate load-bearing capacity after the completion of the main arch ring construction.

**Figure 18:**
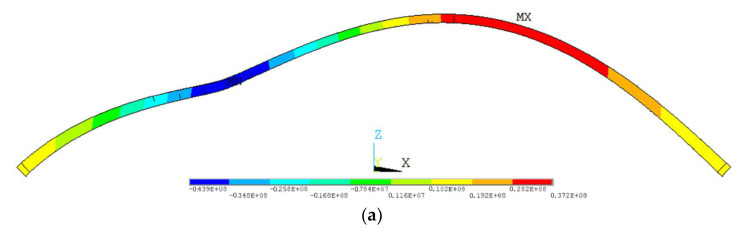

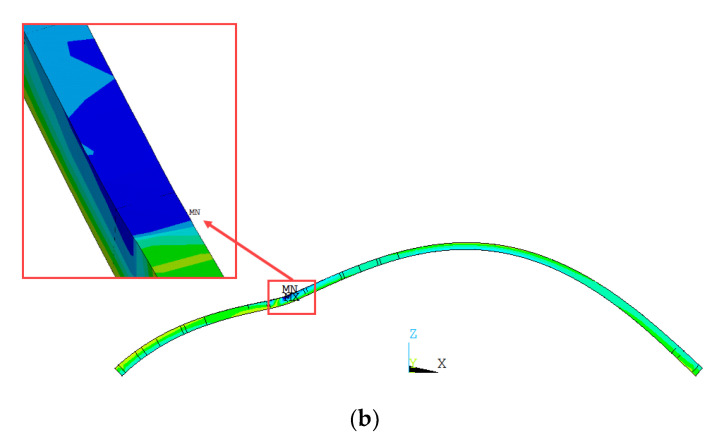
Instability state of the main arch ring under 1/4 span single-point loading condition. (**a**) Deformation diagram of the main arch ring at the point of instability (unit: Pa); (**b**) stress diagram of the main arch ring at the point of instability.

It is worth mentioning that for the 1/4 span single-point loading condition, the ultimate load-bearing capacities of Model 1 and Model 2 are nearly equal. This is because, under this loading condition, the ultimate load-bearing capacity of the main arch ring is controlled by the yield strength of the material near the loading point. During loading, vertical deflection occurs at the loading point. As shown in Figure 18, the concrete of the roof concrete at this location is compressed. Since the compressive stress of Models 1 and 2 are similar after the closure of the main arch ring, and the subsequent loading position and load step length are completely consistent, the roof concrete stress near the loading point of models 1 and 2 simultaneously reaches the material yield strength, causing structural instability.

## 8. Conclusions

In order to investigate the stress evolution of inter-ring and intersegment interfaces during the separate ring and segment casting processes of concrete encasing the main arch ring of a rigid-frame arch bridge, and its impact on the ultimate load capacity, a 1:10 scaled model experiment was conducted on the main arch ring of the Tian’e Longtan Bridge with a main span of 600 m. Structural strain data at key cross-sections throughout the construction process were collected, and the stress evolution laws of inter-ring and intersegment interfaces were analyzed. The ANSYS simulation results and field data were compared, and the effect of uneven stress distribution of encased concrete on the ultimate load capacity under different loading conditions was calculated. The main conclusions are as follows:

(1) The construction order of the three rings of the main arch ring leads to differences in the timing of the floor, web, and roof concrete participating in the overall structural loading. After the closure of the main arch ring encased in concrete, the three rings of concrete exhibit noticeable stress differences. At the same cross-section, the stress level of the web concrete can reach 76% of the floor concrete stress, and the stress level of the roof concrete is less than 20% of the floor concrete stress.
(2) The three rings of concrete exhibit a characteristic of converging stress levels as the construction stage progresses, mainly manifested as an increase in the inter-ring stress ratio of the encased concrete at the same cross-section as the construction stage progresses.
(3) Due to the negative bending moment at the arch crown, casting concrete near the mid-span can significantly reduce the compressive stress level of the floor concrete. When casting the mid-span closure section web and roof concrete, the floor concrete compressive stress, respectively, decreases by 1.46 MPa and 0.89 MPa, with a decrease of 18.02% and 9.36%.
(4) For the same ring of concrete, due to the casting order, there is a difference in the timing of the concrete participating in local structural loading. At the junction of the work planes, there is a certain difference in the stress levels of the concrete on both sides of the interface. After the main arch ring is closed, the intersegment stress ratios of the floor, web, and roof concrete are 60~70%, 40~60%, and within 5%.
(5) ANSYS APDL was used to establish the finite element model of the main arch ring of the model bridge and the stress data of the top chord, inner concrete, and floor concrete on the rigid steel skeleton at the arch crown cross-section and the arch foot cross-section during the entire construction process were compared. The results show that each component’s measured stress and simulation results during the construction process generally match well, and the overall trend is basically consistent. Among them, the maximum relative errors of the simulation results and the measured results for the main steel tube, inner concrete, and floor concrete of the rigid steel skeleton throughout the construction process are 14.42 MPa, 4.09 MPa, and 1.44 MPa, respectively, verifying the correctness of the finite element model.
(6) To study the effect of the uneven stress distribution of encased concrete on the ultimate load capacity of the main arch ring, ANSYS APDL was used to calculate the ultimate load capacity of the two finite element models of the main arch ring encased in concrete under normal construction (Model 1) and encased in concrete in one pour (Model 2) under four loading conditions: mid-span loading, 1/4 span single-point loading, 1/4 span symmetrical loading, and full-span uniform loading. The results show that the loading conditions significantly affect the ultimate load capacity after the closure of the main arch ring. Under mid-span loading and 1/4 span symmetrical loading conditions, compared to Model 2, the ultimate load capacity of Model 1 decreased by 19.16% and 5.23%, respectively, indicating that an uneven stress distribution of encased concrete will reduce the ultimate load capacity after the closure of the main arch ring.

The next steps in the research will be to conduct related segment tests to clarify the stress evolution laws of the inter-ring and intersegment interfaces of ultra-large-span rigid-frame arch bridges under long-term service conditions, to define the load transfer paths between inter-ring and intersegment interfaces, and to conduct parametric analysis to study the impact of uneven stress on the ultimate bearing capacity of the main arch ring external concrete. Simultaneously, in conjunction with multi-objective optimization algorithms, we aim to minimize both the stress difference in the concrete of the floor, web, and roof concrete at the same section and the stress difference between adjacent segments of each ring of concrete. These are considered objective functions. With the maximum compressive stress of the concrete during the entire construction process as the constraint and the length of each working face of each ring of concrete as design variables, we plan to carry out research on the segmental optimization method for the external concrete of rigid-frame arch bridges. This is the task we are about to undertake.

## Figures and Tables

**Table 1:** Main arch ring construction stage division table.

Construction Stage	Construction Content
1# ^1^	Closure of rigid steel frame.
2#	Pouring concrete into the pipe.
3#	Apply 9 times the self-weight of the rigid steel frame on the corresponding loading point.
4#	Apply 9 times the self-weight of the concrete inside the pipe on the corresponding loading point.
5#	Apply the first part of concrete self-weight counterweight on 8 working planes of the floor concrete on the corresponding loading point.
6#	Pour the first part of concrete on 8 working planes of the floor concrete.
7~14#	Apply the required load at each loading point (6 #, 8 #, 10 #, and 12 #) and pouring concrete (7 #, 9 #, 11 #, and 13 #) for the second to fifth part of the 8 working planes of the floor concrete.
15#	Apply the last part of concrete self-weight counterweight on 8 working planes of the floor concrete.
16#	Closure of the floor concrete.
17~26#	Apply the required load at each loading point (16#, 18#, 20#, 22#, and 24#) and pouring concrete (17#, 19#, 21#, 23#, and 25#) for the first to fifth part of the 8 working planes of the web concrete.
27#	Apply the last part of concrete self-weight counterweight on 8 working planes of the web concrete.
28#	Closure of the web concrete.
29~38#	Apply the required load at each loading point (28#, 30#, 32#, 34#, and 36#) and pouring concrete (29#, 31#, 33#, 35#, and 37#) for the first to fifth part of the 8 working planes of the roof concrete.
39#	Apply the last part of concrete self-weight counterweight on 8 working planes of the roof concrete.
40#	Closure of the roof concrete.

^1^ The number + symbol “#” indicates the construction stage number to avoid confusion with other numbers in the text.

**Table 2:** Sensor parameters table.

Sensor Type	Range	Accuracy	Wavelength Conversion Coefficient
Optical Fiber Strain Sensor	±2500 με	0.1% (2.5 με)	0.83
Optical Temperature Sensor	0~100 °C	0.1% (0.1 °C)	0.083

**Table 3:** Construction sequence table of the main arch ring in finite element model 2.

Construction Stage	Construction Content
1#	Closure of rigid steel frame.
2#	Pouring concrete into the pipe.
3#	Apply 9 times the self-weight of the rigid steel frame on the corresponding loading point.
4#	Apply 9 times the self-weight of the concrete inside the pipe on the corresponding loading point.
5#	Pour all external concrete at once.
6#	Apply the required counterweight at each loading point.

**Table 4:** Ultimate bearing capacity results of different models (unit: ton).

	Mid-Span Loading	1/4 Span Single-Point Loading	1/4 Span Symmetric Loading	Full-Span Uniformly Distributed Loading
Model 1	67.1	120.6	279.3	1098.6
Model 2	83.0	120.1	294.7	1307.6
Model 1/Model 2	80.84%	100%	94.77%	84.02%

## Data Availability

The data presented in this study are available from the first and corresponding author upon request. The data is not publicly available due to the policy of the data provider.
